# Supplementary material for: Vegetation enhances curvature-driven dynamics in meandering rivers
Source: Nat Commun. 2024 Mar 4;15:1968. doi: 10.1038/s41467-024-46292-x (PMC10912106; doi:10.1038/s41467-024-46292-x)
Supplement: Supplementary file 1 — Supplementary Information [file 41467_2024_46292_MOESM1_ESM.pdf]

Supplementary Information for

Vegetation enhances curvature-driven dynamics in meandering rivers

Alvise Finotello[1,*], Alessandro Ielpi[2], Mathieu G.A. Lapôtre[3], Eli D. Lazarus[4], Massimiliano Ghinassi[1], Luca Carniello[5], Serena Favaro[5], Davide Tognin[5], Andrea D'Alpaos[1]

1) Department of Geosciences, University of Padua, Padua, IT-35131, Italy.
2) Earth, Environmental and Geographic Sciences, University of British Columbia, Okanagan Campus, Kelowna, BC, V1V 1V7, Canada.
3) Department of Earth and Planetary Sciences, Stanford University, Stanford, CA 94305, USA.
4) Department of Geography and Environmental Science, University of Southampton, Southampton, SO17 1BJ, United Kingdom.
5) Dept. of Civil, Environmental, and Architectural Engineering, University of Padua, Padua, IT-35131, Italy.

*) Corresponding author
Email: alvise.finotello@unipd.it

This file includes:

Supplementary Methods 1 and 2
Supplementary Figures 1 to 14
References for Supplementary Information

Supplementary Methods

1. Principal Component Analysis

Principal Component Analysis (PCA) is a statistical method employed to transform high-dimensional datasets with potentially correlated variables to lower-dimensional subspaces. PCA has already been applied to analyze meandering river planforms in previous studies [1–4]. To prevent PCA from giving comparably more importance to morphometric variables characterized by higher variance, morphometric data in the original data matrix $\mathbf{X} \in \mathbb{R}^{p \times q}$ (where p is the number of original samples and q denotes the number of considered variables) were standardized by subtracting the mean and dividing by the standard deviation of each variable. This ensures that the covariance matrix of $\mathbf{X}$ is equal to its correlation matrix $\mathbf{R}$. PCA then transforms the original data $\mathbf{X}$ into a lower-dimensional subspace represented by $\mathbf{X} = \mathbf{S} \cdot \mathbf{P}' + \mathbf{E}$, where $\mathbf{P}'$ is the projection matrix, $\mathbf{S}$ contains the score vectors ($\mathbf{s_a}$) that define the original data coordinates in the Principal Component (PC) subspace, and $\mathbf{E}$ is the matrix of residuals. The rows of the projection matrix $\mathbf{P}'$ correspond to the loading vectors $\mathbf{a}$ that define the PC subspace.

The eigenvectors of the correlation matrix $\mathbf{R}$ correspond to the axis directions of the PC subspace. Since $\mathbf{R}$ is symmetric, the loading vectors $\mathbf{a}$ are orthogonal by definition and indicate the directions that contain the majority of the variance in the original dataset. Typically, the loading vectors $\mathbf{a}$ are arranged in descending order based on their corresponding eigenvalues, with the assumption that the vectors associated with the largest eigenvalues carry the most informative data.

Several plots are used to visualize and interpret PCA results, such as the score plot (Fig. 3E-G), which shows the score vectors $\mathbf{s_a}$ in the PC subspace, and the biplot (Supplementary Figs. 2 and 3; see also Fig. 3B-D in the Main Text) that combines the principal component loadings and scores to visualize the variables that are mainly responsible for separating distinct data classes. Note that the PC scores in biplots have to be divided by the maximum absolute value of all scores and multiplied by the length of the corresponding loading vectors to fit into the loading space.

2. Curvature-migration lag in meandering rivers

Field and theoretical studies have long suggested a functional relationship between channel curvature and lateral migration in meandering alluvial rivers[5,6].

On the one hand, significant scatter is typically observed in the distribution of C vs. M_{R} data (derived mostly for large, sand-to-gravel bed rivers in vegetated landscapes), which prevents one from relating a given value of M_{R} with a single value of C. On the other hand, however, M_{R} and C are on average closely correlated in a quasi-linear fashion, with higher curvatures corresponding to larger migrations[7–9]. This leads to a strong, first-order control of curvature on meandering river morphodynamics, which is supported by evidence from relatively simple kinematic models reproducing realistic meandering river patterns by linking river lateral migration to weighted convolutions of upstream channel curvature[7,10].

The linear proportionality between C and M_{R} usually holds only for mildly curved channel reaches and breaks down when C>0.3 (this threshold reportedly varies between 0.25 and 0.5, with many authors expressing it as 2<R^*/B^*<4, where $R^* = 1/C^*$ is the meander radius of curvature). Beyond C≅0.3, meander migration tends to saturate due to the growth of hydrodynamic nonlinearities that effectively limit bank erosion, such as saturation of centrifugally driven secondary flows, enhanced secondary outer bank cells, and flow separation at the outer bank[8,11,12]. The relationship between C and the upper values of the M_{R} distribution is even more intricate. These upper values usually increase much more rapidly than the average values as C increases, before sharply decreasing once C exceeds 0.3. This leads to the distribution of (C, M_{R}) pairs to typically be enveloped by a hump-shaped curve, that can be broadly interpreted as representing the maximum potential migration rate[12,13] for a given bend curvature.

Notably, the above-described proportionalities emerge more clearly once the spatial lag between the points of maximum curvature (i.e., meander apexes) and the loci of maximum lateral migration[7–9,14,15] is accounted for. Such a lag arises as a direct result of the flow-field structure along sinuous erodible channels, and implies that lateral migration (M^*) typically lags behind relative to local curvature (C^*) (Supplementary Fig. 11). Recent observations[7,9] demonstrated that the curvature-

migration lag ultimately determines the migration path of river meanders, and is therefore critical to be accounted for in order to gain information regarding the relation between local channel curvature and lateral migration rates.

For meandering rivers falling within the so-called sub-resonant morphodynamic regime – which is the most common regime and occurs for values of the channel width-to-depth ratio (β) smaller than a critical resonant value (β_{R}) (refs. [16–18]) - peak bank erosion occurs downstream of the point of maximum channel curvature[19]. This leads to the formation of upstream-skewed, downstream-migrating meander bends. However, fluvial-meander theory suggests that even a super-resonant morphodynamic regime (β>β_{R}) can be achieved, whereby the erosion peak occurs upstream of the bend apex and lead to the formation of downstream-skewed, upstream migrating meanders[17,18,20]. Note that both sub-resonant and super-resonant conditions are encountered in nature, sometimes even along the same river reach[17] (Supplementary Fig. 11). Hence, the curvature-migration lag is not necessarily constant but rather a function of the local morphodynamic regime and related meander features[16]. Moreover, the lag is likely to vary between different river systems depending on a range of factors (e.g., underlying geology, sediment characteristics, and environmental conditions). This notwithstanding, previous analyses suggested that the along-channel distance between peak curvatures and migrations is fairly consistent across a wide range of climates and geological settings, typically attaining values ranging between 2 to 3 channel widths[7,9,15,21]. Our data confirm the above. It is clear from measurements of local migration rates in time-lapse satellite images that the pattern of river migration rate closely follows that of the local curvature, with a roughly constant phase lag between the two (see an example from the actively migrating Rio Horton in Supplementary Fig. 11). Moreover, as illustrated by Supplementary Fig. 14, the reach-averaged curvature-migration lag (Δ_{CM}^* [-]) consistently scales with river width across a wide range of environmental and climatic settings (as typified by distinct riparian vegetation density), with values just slightly larger than two channel widths. Thus, in order to account for the spatial lag between curvature and migration maxima, in our analyses we first computed M^* and C^* separately (see Methods in the Main Text), and then shifted the M^* signal upstream by a length Δ_{CM}^*, so that we could study the M^* vs. C^* functional relationship in a physically sound fashion.

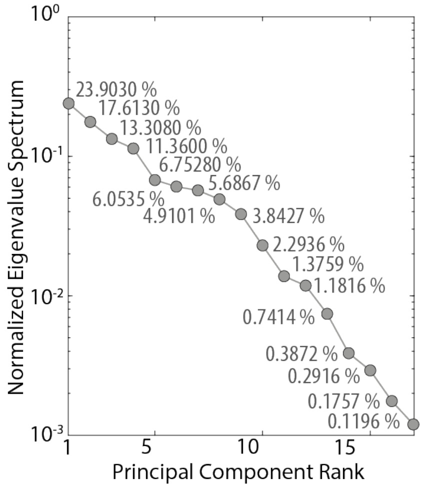

Supplementary Figure 1. Principal component (PC) normalized eigenvalue spectrum for the subset of variables shown in Fig.3 in the Main Text. The percentage of spectral power accounted for by each PC is also reported.

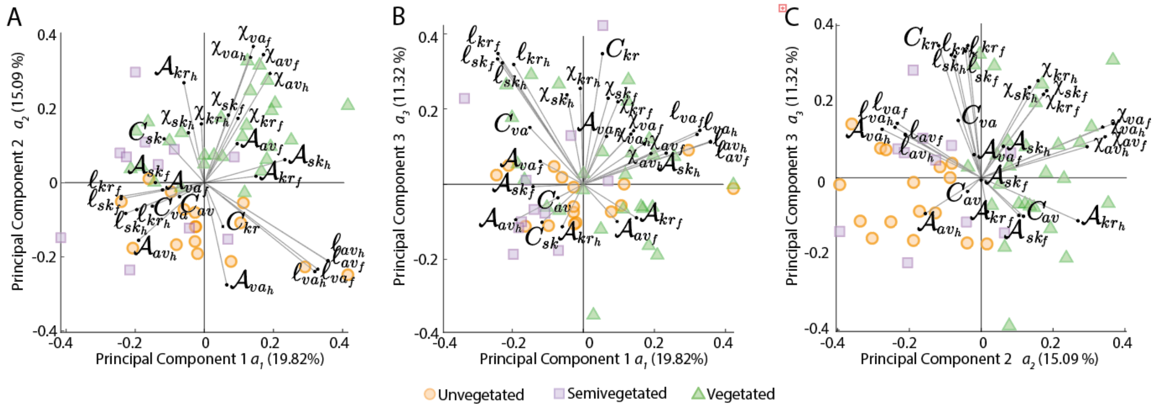

Supplementary Figure 2. Principal Component Analysis (PCA) of meandering river planforms performed by retaining all the available morphometric variables (Table 1). Each panel contains a distinct biplot of the Principal Component (PC) loadings and scores for the first three PCs (a_1, a_2, and a_3). PC loadings correspond to the correlation coefficients defining PCs, while scores are the projections of the original data into the PC space. In order to fit in the loading space, PC scores are divided by the maximum absolute value of all scores and multiplied by the length of the loading vectors. The percent of variance explained by each PC is reported along corresponding axes. The reader is referred to Table 1 in the Main Text for symbology meaning.

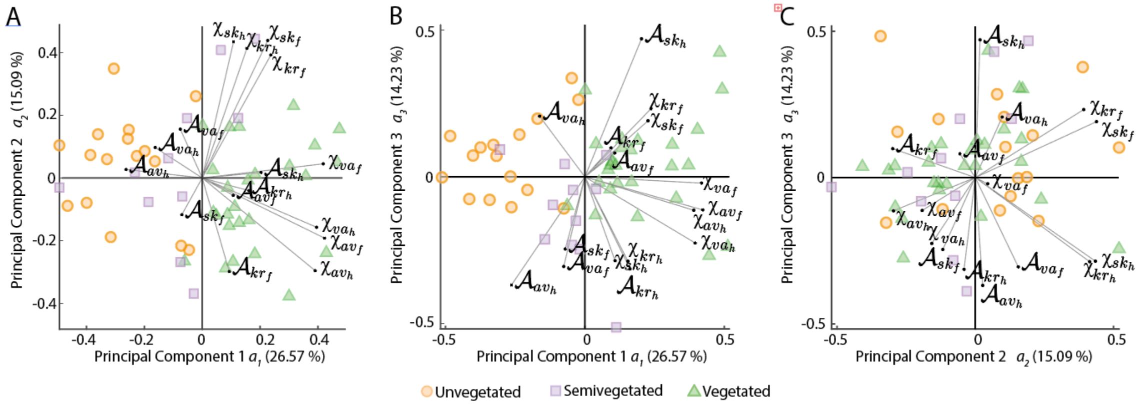

Supplementary Figure 3. Principal Component Analysis (PCA) of meandering river planforms performed by retaining only the morphometric variables that are independent of meander width, that is, those related to meander sinuosity (χ) and asymmetry ($\mathcal{A}$) (Table 1). Each panel contains a distinct biplot of the Principal Component (PC) loadings and scores for the first three PCs (a_1, a_2, and a_3). PC loadings correspond to the correlation coefficients defining PCs, while scores are the projections of the original data into the PC space. In order to fit in the loading space, PC scores are divided by the maximum absolute value of all scores and multiplied by the length of the loading vectors. The percent of variance explained by each PC is reported along corresponding axes. The reader is referred to Table 1 in the Main Text for symbology meaning.

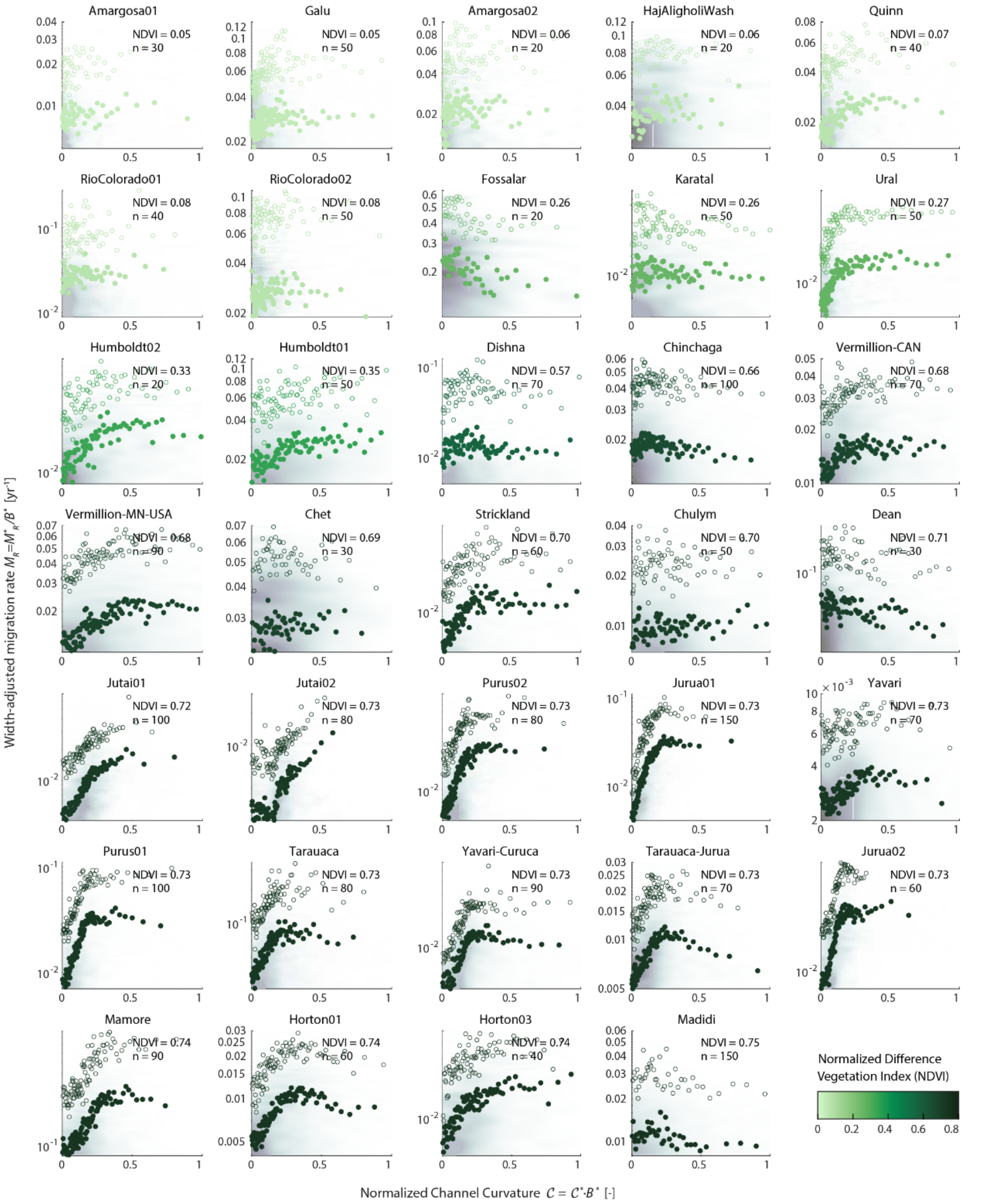

Supplementary Figure 4. Width-adjusted migration rates ($M_{\mathrm{R}} = M^*_{\mathrm{R}}/B^*$ [yr^{-1}]), corrected for the reach-averaged spatial lag between maximum migration and curvature (Δ^*_{CM}), are plotted against the width-normalized curvature ($\mathcal{C} = C^* \cdot B^*$ [-]) for each reach. Filled and empty dots represent the binned 50th and 95th percentiles of the M_{R} vs. $\mathcal{C}$ distribution, respectively, obtained from a set of *n* data points specified in each panel. Dots are colored based on the Normalize Difference Vegetation Index (NDVI) computed for each river floodplain. Shaded areas in the background represent the bidimensional kernel density estimates of the data, with darker colors denoting higher kernel densities. NDVI values increase from left to right and from top to bottom.

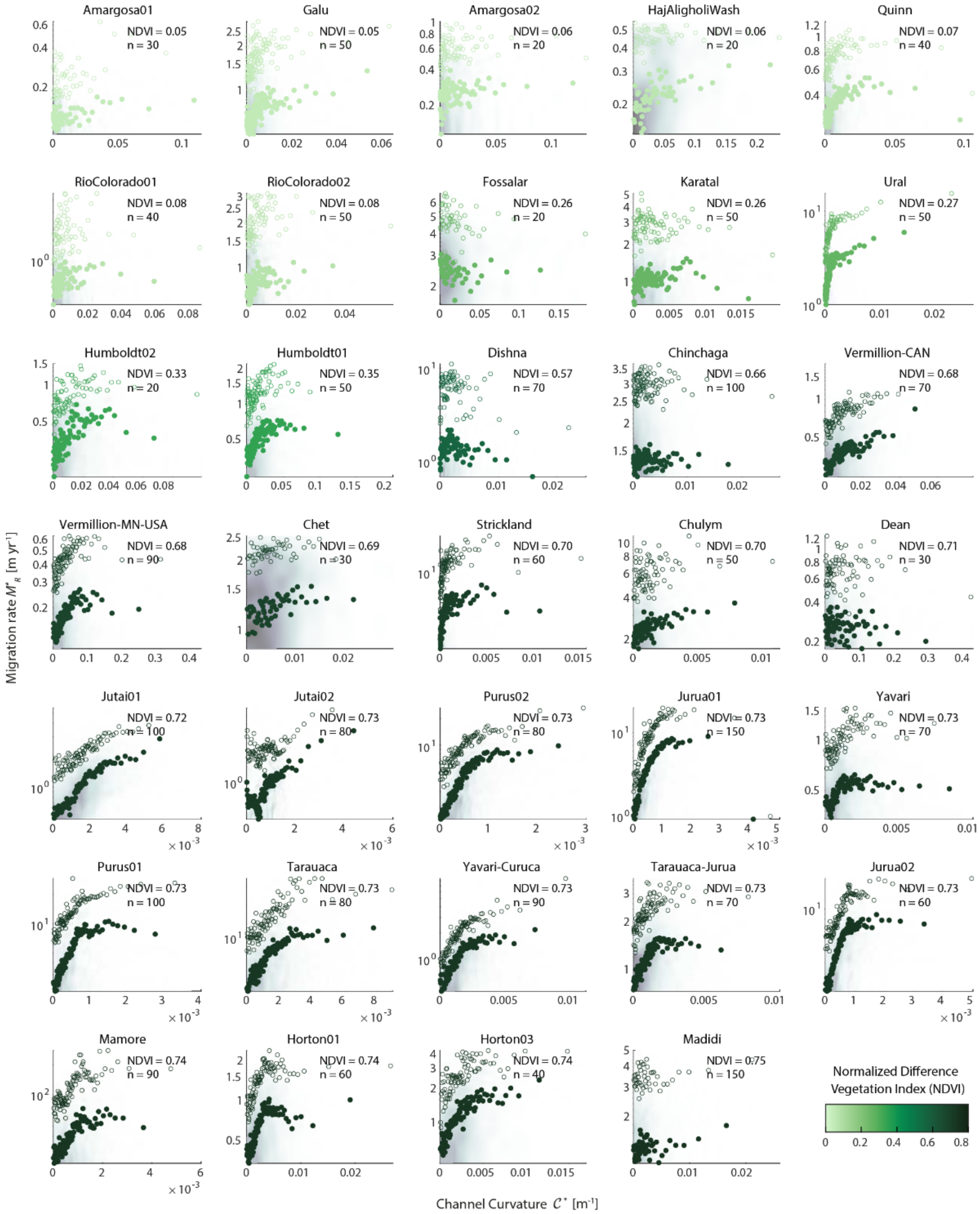

Supplementary Figure 5. River lateral migration rates (M^*_{R} [m yr^{-1}]), corrected for the reach-averaged spatial lag between maximum migration and curvature (Δ^*_{CM}), are plotted against river curvature (C^* [m^{-1}]) for each reach. Filled and empty dots represent the binned 50th and 95th percentiles of the M^*_{R}vs. C^* distribution, respectively, obtained from a set of *n* data points specified in each panel. Dots are colored based on the Normalize Difference Vegetation Index (NDVI) computed for each river floodplain. Shaded areas in the background represent the bidimensional kernel density estimates of the data, with darker colors denoting higher kernel densities. NDVI values increase from left to right and from top to bottom.

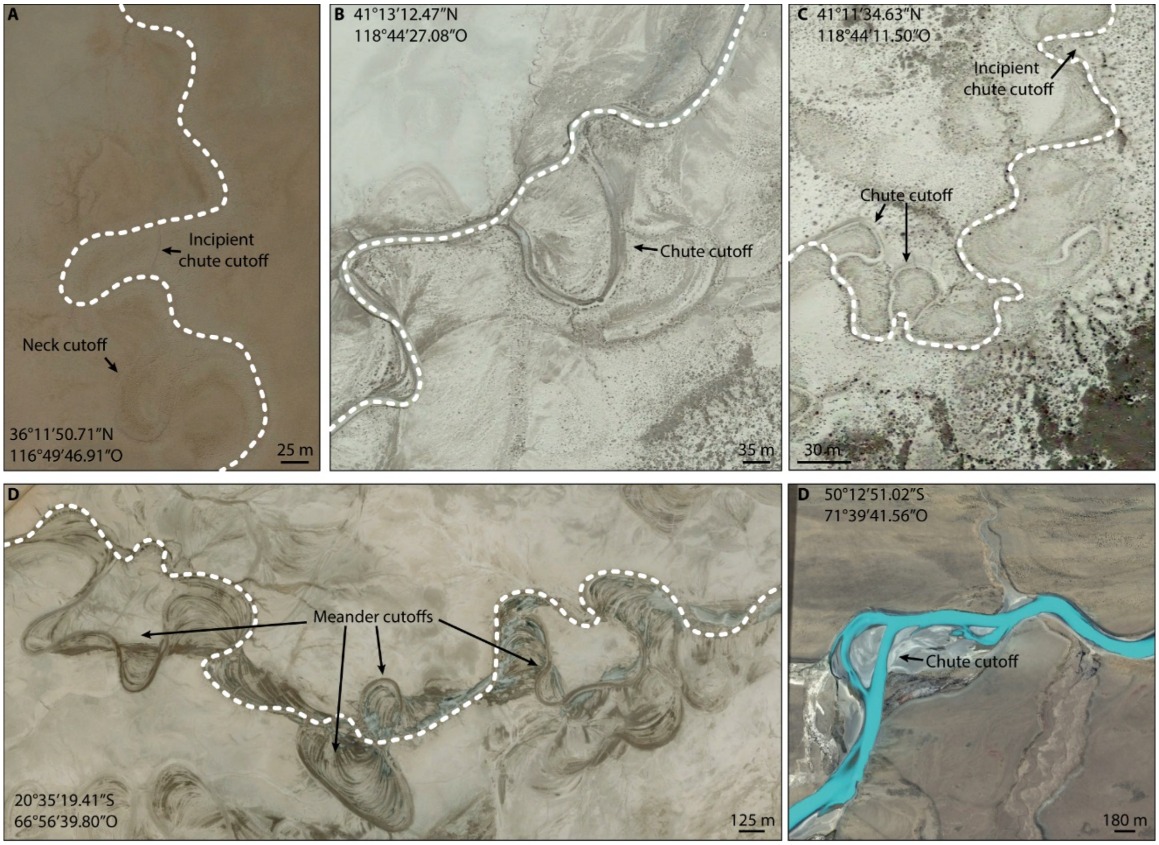

Supplementary Figure 6. Examples of meander cutoffs in unvegetated rivers. (A) Amargosa River, California, USA (Map data: Google, Maxar technologies, CNES/ Airbus; image date: 2015/06/09). (B,C) Quinn River, Nevada, USA (Map data: Google, Landsat, Copernicus; image data: 2013/07/21). (D) Rio Colorado, Salar de Uyuni, Bolivia (Map data: Google, Maxar technologies; image date: 2004/02/11). (E) Rio Santa Cruz, Los Glaciares National Park, Argentina (Map data: Maxar technologies, CNES/ Airbus; image date: 2015/11/21).

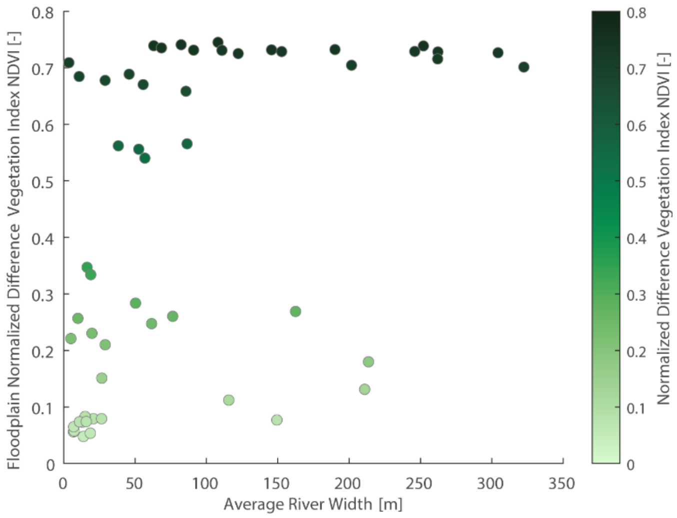

Supplementary Figure 7. Scatter plot of floodplain Normalized Difference Vegetation Index (NDVI) vs. river width for all the rivers in the analyzed dataset.

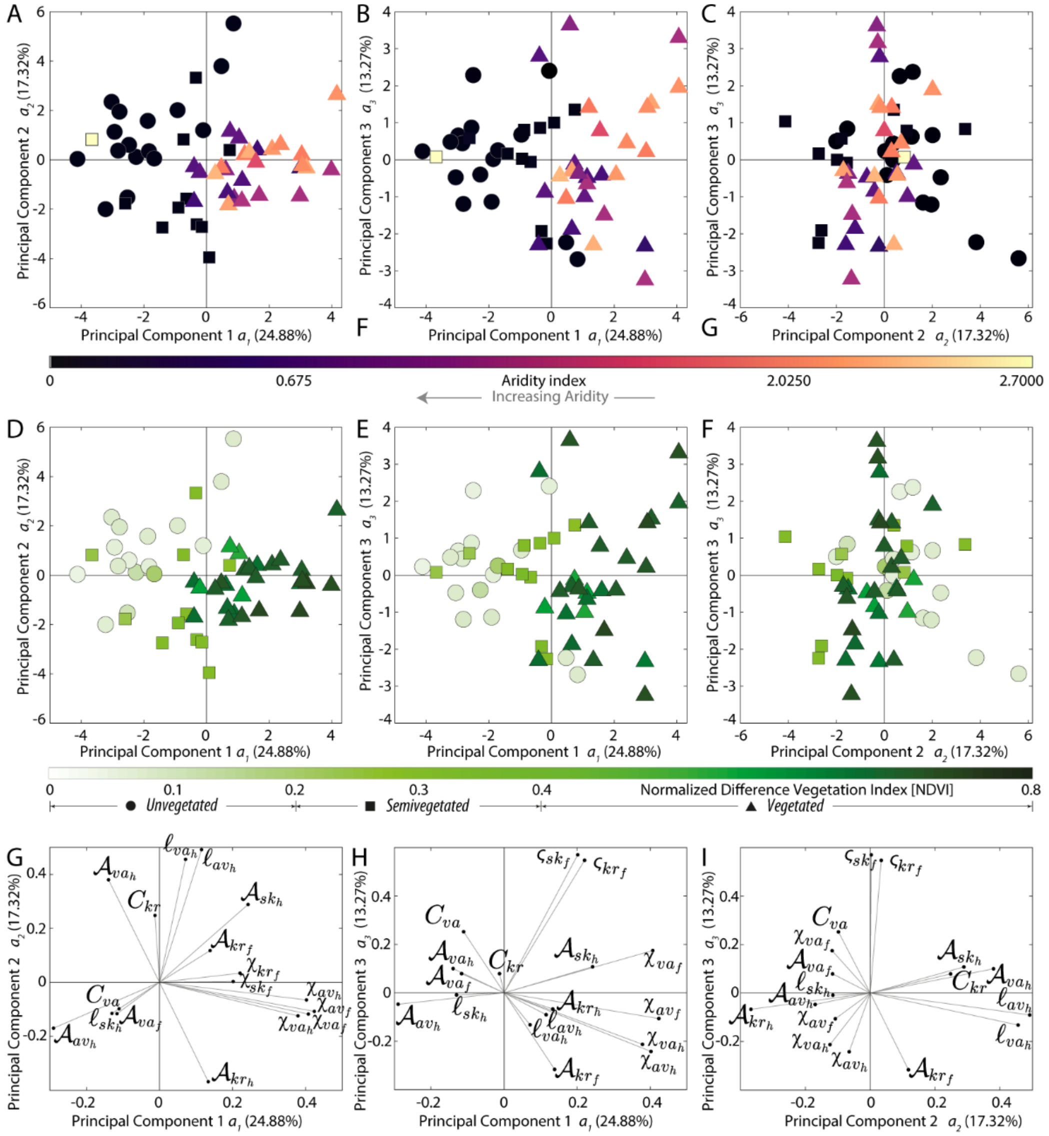

Supplementary Figure 8. Principal Component Analysis (PCA) of meandering river planform morphometrics. (A to G) Score plots for the first three PCs (a_1, a_2, and a_3) resulting from PCA. Colors represent the Aridity Index (A,B,C) and the Normalized Difference Vegetation Index (D,E,F) for each studies river, whereas dots, squares, and triangles denote unvegetated, semi-vegetated, and vegetated rivers, respectively. (G,H,I) Loading plots for the first three PCs (a_1, a_2, and a_3) resulting from PCA. The suite of morphometric variables used to carry out PCA is the same reported in Fig. 3 in the Main Text.

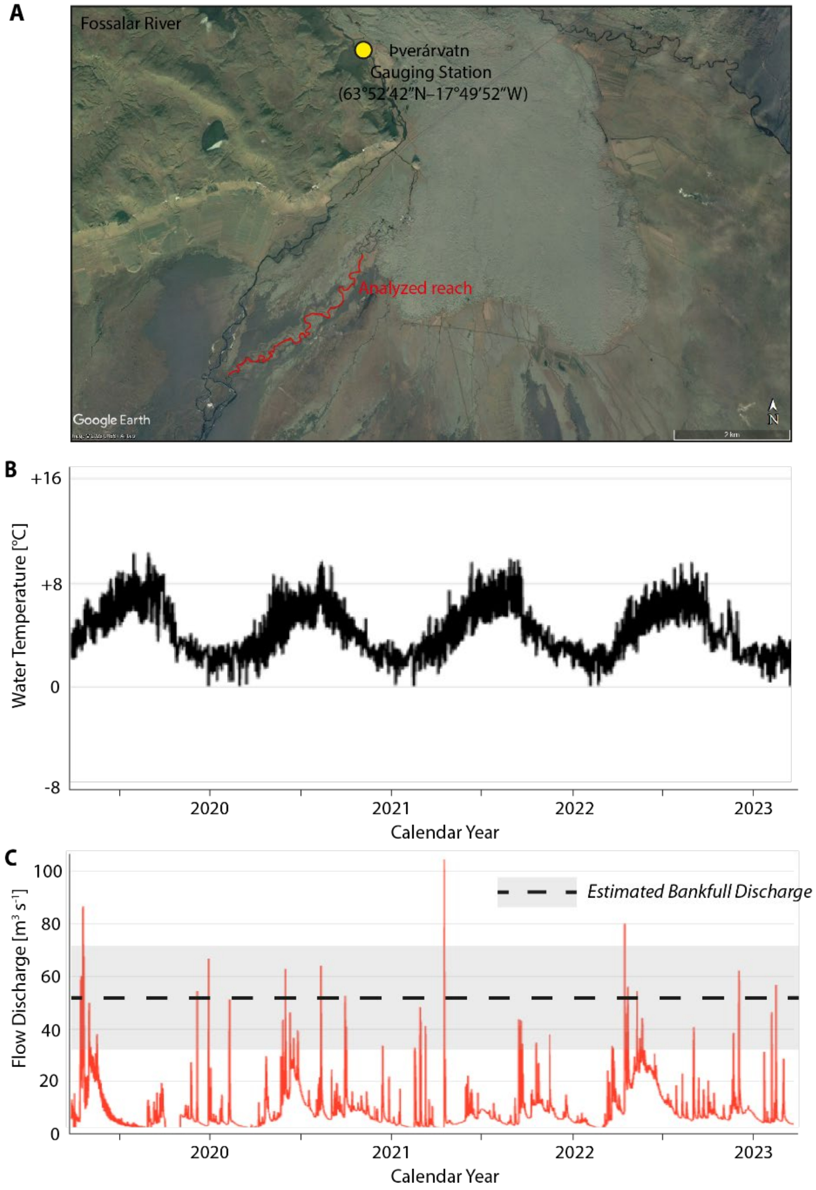

Supplementary Figure 9. Geomorphology and hydrology of the Fossalar River (Iceland). (A) Aerial image of the study area. The analyzed reach of the Fossalar River, corresponding to the left-side tributary described in Ielpi[22], is highlighted in red. The yellow dot denotes the position of the Þverárvatn gauging station, along the main trunk of the river (image © Google, CNES/Airbus – Maxar technologies). (B,C) Multiyear record of water temperature (B) and flow discharge (C) data for the main trunk of the Fossalar River as derived from the Iceland Meteorological Office website for the Þverárvatn gauging station (http://vmkerfi.vedur.is/vatn/vdv.php/historical/612). The dashed line represents the estimated bankfull discharge for the main trunk of the Fossalar River based on the empirical relationship of Bjerklie[23], with a shaded gray area denoting the 95% prediction interval.

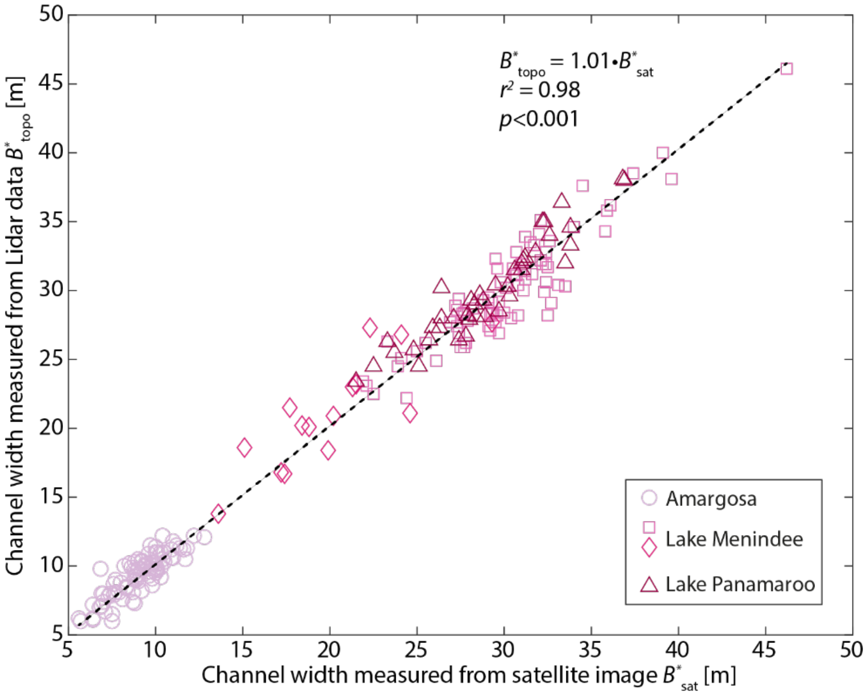

Supplementary Figure 10. Comparison between river width measured from satellite images (B^*_{sat} [m]) vs. river width measured from high-resolution topographic data (B^*_{topo} [m]) available at 0.5 m resolution from the open topography portal (https://portal.opentopography.org/) for the Amargosa River (California, USA) and at 1m resolution from the Australia Government's Elevation Information System, ELVIS (https://elevation.fsdf.org.au/) for the meandering streamflow systems found in the Menindee and Panamaroo lakes (Darling River basin, New South Wales, Australia). The dashed line represents the linear regression of data points. Linear-regression equation, together with R-squared ($\boldsymbol{r^2}$) and *p*-value are also reported.

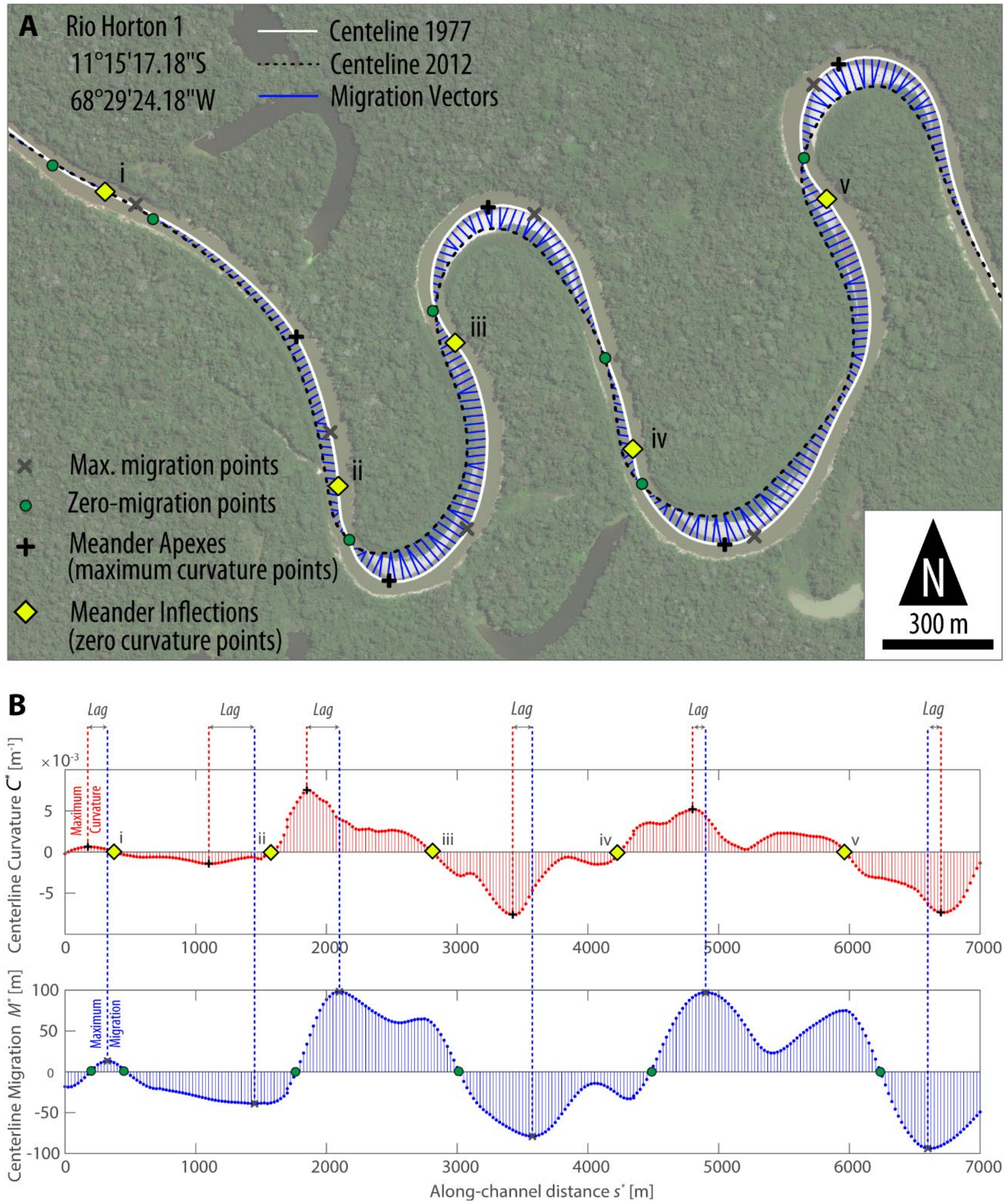

Supplementary Figure 11. Meander morphologies and dynamics in the sinuous Rio Horton (Bolivia). (A) Comparison of river centerlines in 1977 (continuous white line) and 2012 (dashed black line). The positions of meander apexes (i.e., maximum curvature points), meander inflections (zero-curvature points), maximum migration points, and zero-migration points are also highlighted, along with migration vectors computed through dynamic time warping. (B) Plots of river centerline curvature and migration as a function of streamwise curvilinear coordinate. The symbols for meander apexes and inflections, as well as for maximum and zero-migration points are consistent with those reported in panel (A). Meander inflections are also numbered for visual reference. The spatial lags between loci of maximum migration and maximum curvature are also highlighted.

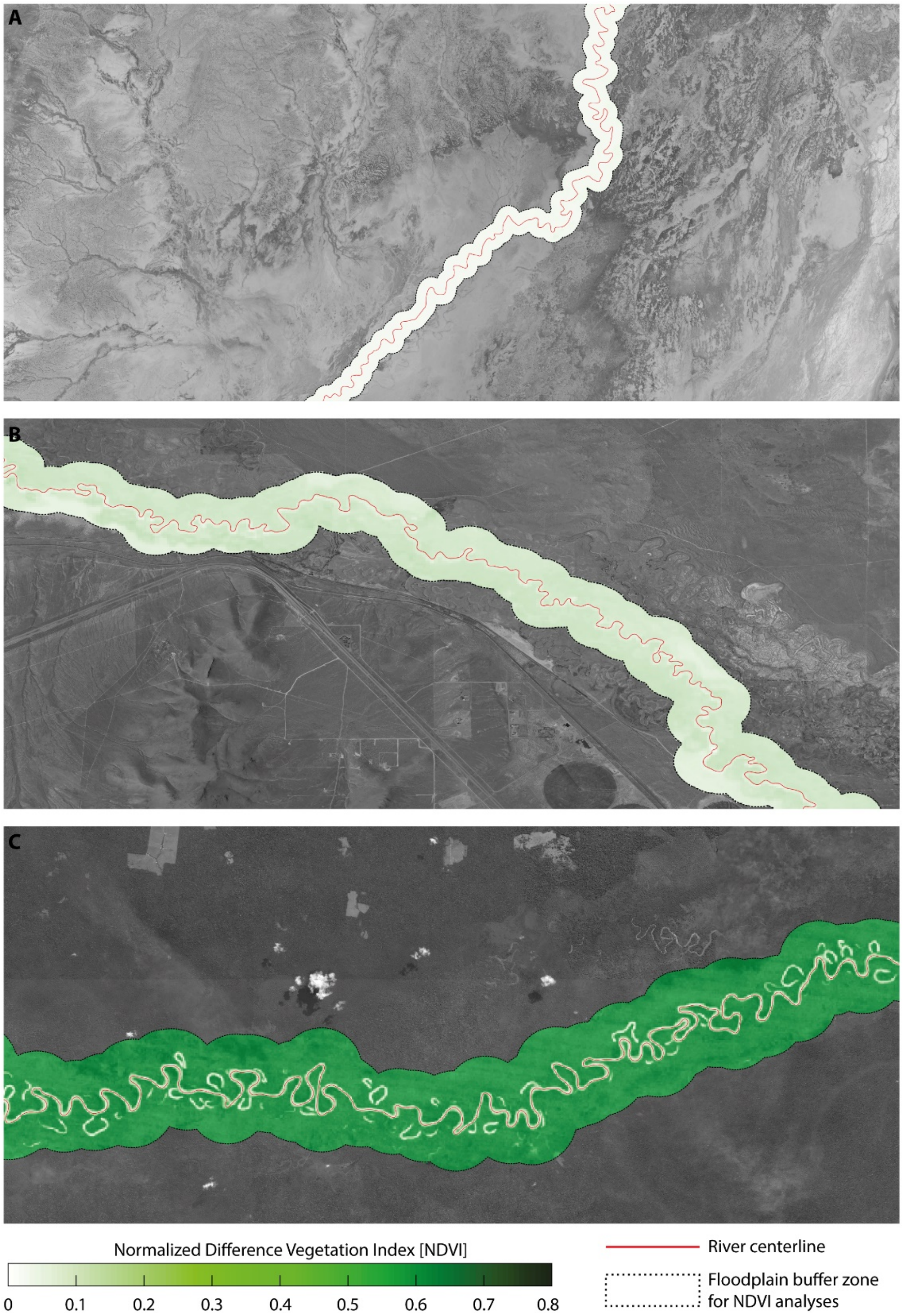

Supplementary Figure 12. Example of river floodplain Normalized Difference Vegetation Index (NDVI) calculation. (A) Quin River, Nevada, USA (image © Google, Maxar Technologies). (B) Humboldt River, Nevada, USA (image © Google, Landsat/Copernicus). (C) Rio Horton, Bolivia (image © Google, Landsat/Copernicus). Red lines represent river centerlines, whereas the floodplain area used to compute the reach-average NDVI is bounded by black dotted lines and colored according to the local NDVI value (see Methods in the main text for details regarding NDVI calculations).

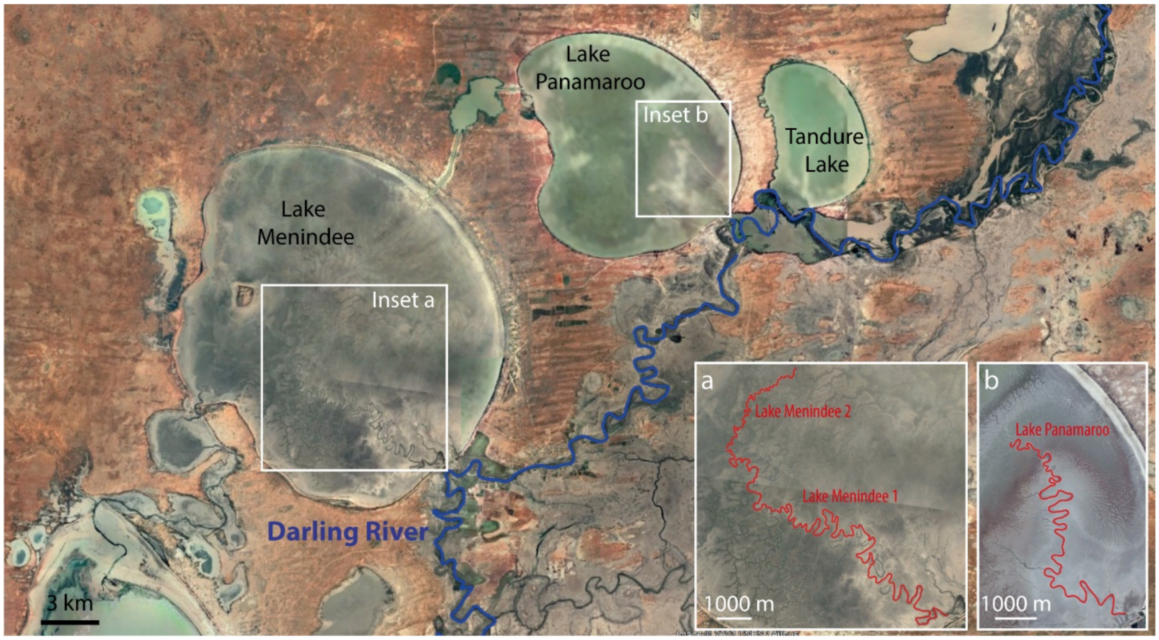

Supplementary Figure 13. Aerial view of Lakes Menindee and Panamaroo (New South Wales, Australia). The lakes are part of the so-called “Menindee Lakes system”, consisting of 9 large, relatively shallow, partially interconnected lakes that originate from the Darling River about 200 km upstream of the junction with the River Murray. Insets *a* and *b* show a closeup view of the meandering streams found in Lake Menindee and Lake Panamaroo, respectively, that were analyzed in the present study (image © Google, Maxar Technologies).

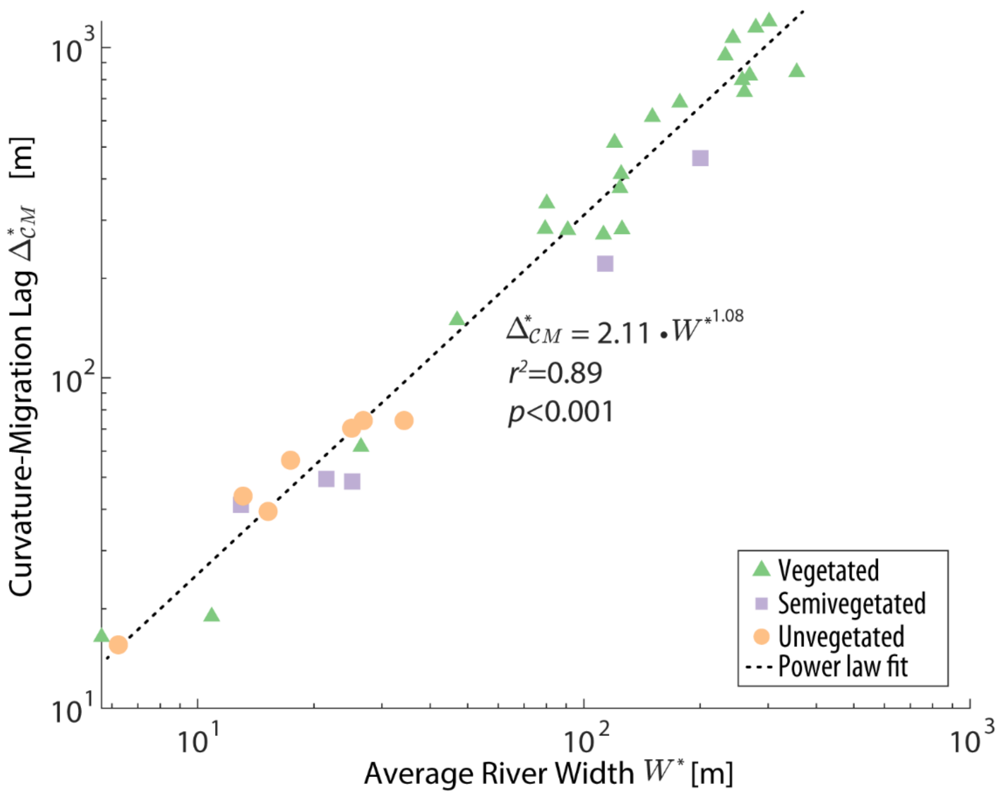

Supplementary Figure 14. Scatter plot of average river width vs. reach-average values of the curvature-migration lag (Δ^*_{CM}) for all the rivers in the analyzed dataset. The dashed line represents linear regression on log-transformed data. data points. Power-law regression coefficients, together with R-Squared (r^2) and p-value are also reported.

Supplementary References

1. Finotello, A., D'Alpaos, A., Bogoni, M., Ghinassi, M. & Lanzoni, S. Remotely-sensed planform morphologies reveal fluvial and tidal nature of meandering channels. *Sci. Rep.* 10, 1–13 (2020).

2. Howard, A. D. & Hemberger, A. T. Multivariate characterization of meandering. *Geomorphology* 4, 161–186 (1991).

3. Frascati, A. & Lanzoni, S. Morphodynamic regime and long-term evolution of meandering rivers. *J. Geophys. Res. Earth Surf.* 114, 1–12 (2009).

4. Bogoni, M., Putti, M. & Lanzoni, S. Modeling meander morphodynamics over self-formed heterogeneous floodplains. *Water Resour. Res.* 53, 5137–5157 (2017).

5. Hickin, E. J. & Nanson, G. C. The Character of Channel Migration on the Beatton River, Northeast British Columbia, Canada. *Geol. Soc. Am. Bull.* 86, 487 (1975).

6. Nanson, G. C. & Hickin, E. J. Channel Migration and Incision on the Beatton River. *J. Hydraul. Eng.* 109, 327–337 (1983).

7. Sylvester, Z., Durkin, P., Covault, J. A. & Sharman, G. R. High curvatures drive river meandering. *Geology* 47, e486–e486 (2019).

8. Finotello, A., D'Alpaos, A., Lazarus, E. D. & Lanzoni, S. High curvatures drive river meandering: COMMENT. *Geology* 47, e485–e485 (2019).

9. Donovan, M., Belmont, P. & Sylvester, Z. Evaluating the Relationship Between Meander-Bend Curvature, Sediment Supply, and Migration Rates. *J. Geophys. Res. Earth Surf.* 126, 1–20 (2021).

10. Howard, A. D. & Knutson, T. R. Sufficient conditions for river meandering: A simulation approach. *Water Resour. Res.* 20, 1659–1667 (1984).

11. Blanckaert, K. Hydrodynamic processes in sharp meander bends and their morphological implications. *J. Geophys. Res. Earth Surf.* 116, 1–22 (2011).

12. Hooke, J. M. River Meandering. in *Treatise on Geomorphology* (eds. Wohl, E. & Schroder) vol. 9 260–288 (Elsevier, 2013).

13. Finotello, A. *et al.* Field migration rates of tidal meanders recapitulate fluvial morphodynamics. *Proc. Natl. Acad. Sci.* 115, 1463–1468 (2018).

14. Tambroni, N. & Seminara, G. Are inlets responsible for the morphological degradation of Venice Lagoon? *J. Geophys. Res. Earth Surf.* 111, 1–19 (2006).

15. Li, Y. & Limaye, A. B. Testing Predictions for Migration of Meandering Rivers: Fit for a Curvature-Based Model Depends on Streamwise Location and Timescale. *J. Geophys. Res. Earth Surf.* 127, 1–22 (2022).

16. Seminara, G. Meanders. *J. Fluid Mech.* 554, 271–297 (2006).

17. Zolezzi, G. & Seminara, G. Downstream and upstream influence in river meandering. Part 1. General theory and application overdeepening. *J. Fluid Mech.* 438, 183–211 (2001).

18. Seminara, G., Zolezzi, G., Tubino, M. & Zardi, D. Downstream and upstream influence in river meandering. Part 2. Planimetric development. *J. Fluid Mech.* 438, 213–230 (2001).

19. Dietrich, W. E., Smith, J. D. & Dunne, T. Flow and sediment transport in a sand bedded meander. *J. Geol.* 87, 305–315 (1979).

20. Blondeaux, P. & Seminara, G. A unified bar–bend theory of river meanders. *J. Fluid Mech.* 157, 449–470 (1985).

21. Hooke, R. L. B. Distribution of Sediment Transport and Shear Stress in a Meander Bend. *J. Geol.* 83, 543–565 (1975).

22. Ielpi, A. Controls on sinuosity in the sparsely vegetated Fossálar River, southern Iceland. *Geomorphology* 286, 93–109 (2017).

23. Bjerklie, D. M. Estimating the bankfull velocity and discharge for rivers using remotely sensed river morphology information. *J. Hydrol.* 341, 144–155 (2007).
